# Antiviral Effects of Hydroxychloroquine and Type I Interferon on In Vitro Fatal Feline Coronavirus Infection

**DOI:** 10.3390/v12050576

**Published:** 2020-05-24

**Authors:** Tomomi Takano, Kumi Satoh, Tomoyoshi Doki, Taishi Tanabe, Tsutomu Hohdatsu

**Affiliations:** 1 Laboratory of Veterinary Infectious Disease, School of Veterinary Medicine, Kitasato University, Towada, Aomori 034-8628, Japan; kumi@vmas.kitasato-u.ac.jp (K.S.); doki@vmas.kitasato-u.ac.jp (T.D.); hohdatsu@vmas.kitasato-u.ac.jp (T.H.); 2 Laboratory of Veterinary Microbiology, School of Veterinary Medicine, Kitasato University, Towada, Aomori 034-8628, Japan; tanabe@vmas.kitasato-u.ac.jp

**Keywords:** feline coronavirus, feline infectious peritonitis, hydroxychloroquine, interferon

## Abstract

Feline infectious peritonitis (FIP) is a viral disease with a high morbidity and mortality by the FIP virus (FIPV, virulent feline coronavirus). Several antiviral drugs for FIP have been identified, but many of these are expensive and not available in veterinary medicine. Hydroxychloroquine (HCQ) is a drug approved by several countries to treat malaria and immune-mediated diseases in humans, and its antiviral effects on other viral infections (e.g., SARS-CoV-2, dengue virus) have been confirmed. We investigated whether HCQ in association with interferon-ω (IFN-ω) is effective for FIPV in vitro. A total of 100 μM of HCQ significantly inhibited the replication of types I and II FIPV. Interestingly, the combination of 100 μM of HCQ and 10^4^ U/mL of recombinant feline IFN-ω (rfIFN-ω, veterinary registered drug) increased its antiviral activity against type I FIPV infection. Our study suggested that HCQ and rfIFN-ω are applicable for treatment of FIP. Further clinical studies are needed to verify the combination of HCQ and rIFN-ω will be effective and safe treatment for cats with FIP.

## 1. Introduction

Coronaviruses are single-stranded positive-sense RNA viruses in the subfamily *Orthocoronavirinae* of the family *Coronaviridae* [1]. Coronaviruses are important pathogens causing life-threatening infectious disease in mammals and birds [2]. In humans, outbreaks of the 2019 novel coronavirus (2019-nCoV, official name is Severe Acute Respiratory Syndrome-related Coronavirus 2: SARS-CoV-2) occurred worldwide [3].

Feline infectious peritonitis (FIP) is a fatal, immune-mediated disease caused by feline coronavirus (FCoV) [4]. FCoV is a member of the species Alphacoronavirus-1, genus *Alphacoronavirus*, in the subfamily *Orthocoronavirinae*. It is divided into two serotypes based on the amino acid sequence of the spike (S) protein, serotype I FCoV, and serotype II FCoV [5]. The majority of FCoV infections are subclinical (avirulent FCoV is known as feline enteric coronavirus: FECV; type I and type II FECV) [6]. However, several mutations occurred in the S protein, leading to development of the virulent type called feline infectious peritonitis virus (FIP virus, FIPV; type I and type II FIPV) [7,8]. FIPV infection typically causes a fatal disease in cats known as FIP. The hallmark pathological findings of FIP in cats are serous fluid in peritoneal and pleural cavities, and pyogranulomatous lesions in the internal organs and brain [4].

Antiviral drugs against FIP have been investigated for decades. In the past few years, several promising antivirals have been developed. GS-441524 (active triphosphate forms of Remdesivir) and GC-364 (3C-like protease inhibitor) have demonstrated great promise in the treatment of cats with FIP [9,10]. However, these drugs are not practically used for reasons of unavailable in veterinary medicine. Moreover, they will probably be expensive and can lead to antiviral resistance mutation [9,10].

We previously demonstrated that the anti-malarial drug, chloroquine (CQ), has anti-FIPV effects [11]. The clinical score of chloroquine-treatment groups was better than in the chloroquine-untreated group. On the other hand, it may increase the levels of alanine aminotransferase in cats with FIP. Hydroxychloroquine (HCQ), a chloroquine derivative, is much less toxic than CQ in mouse, rat, and dog [12]. HCQ was suggested to be effective for other viral infections (SARS-CoV-2 and dengue virus) [13,14]. HCQ is not expensive and widely available. However, the antiviral activity of HCQ on FIPV infection has not been investigated.

Type I interferon (IFN) is an important cytokine for host defense against viral infections [15]. Feline IFN-ω(fIFN-ω) has been identified as feline type I IFN [16]. Recombinant fIFN-ω protein (rfIFN-ω) is a drug approved for veterinary medicine, and is used to treat feline calicivirus infection, feline immunodeficiency virus infection, feline leukemia virus infection, and canine parvovirus-2 infection [17,18,19]. On the other hand, different results have been reported regarding the efficacy of rIFN-ω for FIP [20,21]. Ishida et al. demonstrated rfIFN-ω could improve the clinical status of cats with FIP. On the other hand, Ritz et al. showed no clinical effect of rfIFN-ω for FIP by randomized controlled trial. i.e., there is room to discuss the use of rfIFN-ω for treatment of FIP. It has been reported that 7a protein of FIPV inhibits the antiviral activity of type I IFN [22]. Therefore, it is necessary to combine a drug improving the action of rfIFN-ω to treat FIP. HCQ/rfIFN-ω combination therapy may be a safe and effective treatment method for cats.

In this study, we examined the antiviral effects of HCQ alone or in combination with rfIFN-ω on both serotypes of FIPV, and found that the antiviral effects of HCQ against FIPV infection were increased by rfIFN-ω.

## 2. Materials and Methods

### 2.1. Cell Cultures and Viruses

*Felis catus* whole fetus (fcwf)-4 cells (kindly supplied by Dr. M. C. Horzinek of Universiteit Utrecht) were grown in Eagles’ MEM containing 50% Leibovitz’s L-15 medium, 5% fetal calf serum (FCS), 100 U/mL of penicillin, and 100 μg/mL of streptomycin. The maintenance medium was the same composition as the growth medium except for the concentration of FCS (2%). The type I FCoV KU-2 strain (FIPV-I KU-2) was isolated in our laboratory. The type I FCoV UCD-1 strain (FIPV-I UCD-1) and the type I FIPV UCD-4 strain (FIPV-I UCD-4) were kindly supplied by Dr. J. K. Yamamoto from the University of Florida. The type II FCoV WSU 79-1146 strain (FIPV-II 79-1146) was kindly provided by Dr. M. C. Horzinek of Universiteit Utrecht. These viruses were grown in fcwf-4 cells at 37 °C.

### 2.2. Compounds

CQ (chloroquine diphosphate) and HCQ (hydroxychloroquine sulfate) were purchased from Sigma Aldrich Japan (Tokyo, Japan). rfIFN-ω (INTERCAT^TM^) was purchased from TORAY (Tokyo, Japan). CQ and HCQ were dissolved in maintenance medium as 10 mM stock. rfIFN-ω was dissolved in maintenance medium as 10^6^ U/mL stock. On the day of the experiments, these compounds were diluted to the desired concentrations in maintenance medium.

### 2.3. Cytotoxic Effects of Compounds

The fcwf-4 cells were seeded on 96-well plates. The compounds were added in triplicate to the wells. After incubation for defined periods, the culture supernatants were removed, WST-8 solution (Kishida Chemical, Japan) was added, and the cells were returned to the incubator for 1 h. The absorbance of formazan produced was measured at 450 nm using a 96-well spectrophotometric plate reader, as described by the manufacturer. Percentage cell viability was calculated using the following formula: Cell viability (%) = [(OD of compound-untreated cells - compound-treated cells)/(OD of compound-untreated cells)] × 100. The 50% cytotoxicity concentration (CC_50_) was defined as the cytotoxic concentration of each compound that reduced the absorbance of treated cells to 50% when compared with that of the untreated cells.

### 2.4. Antiviral Effects of Compounds

Confluent fcwf-4 cell monolayers were cultured in medium with or without compounds at the indicated concentrations in 24-well multi-plates at 37 °C for 24 h or 1 h. Cells were washed and the virus (MOI 0.01) was adsorbed into the cells at 37 °C for 1 h. After washing, cells were cultured in 1.5% carboxymethyl cellulose (CMC)-MEM or MEM with or without compounds. In the case of cells cultured in CMC-MEM, the cell monolayers were incubated at 37 ºC for 48 h, fixed, and stained with 1% crystal violet solution containing 10% buffered formalin, and the resulting plaques were then counted. The percentage of the decrease or increase in plaques was calculated using the following formula: Percentage of the plaque reduction (%) = [(plaque number of compound-treated cells)/(plaque number of compound-untreated cells)] × 100. The EC_50_ was defined as the effective concentration of compounds that reduced the virus titer in the culture supernatant of infected cells to 50% when compared with that of the virus control. In the case of cells cultured in MEM, the culture supernatants were collected 48 h post-infection, and virus titers were measured by the TCID_50_ assay.

### 2.5. Immunofluorecence Assay (IFA)

The nucleocapsid (N) protein levels of FIPV-infected fcwf-4 cells were determined by an immunofluorescence assay (IFA), as described previously [23]. Briefly, FIPV-infected cells were washed PBS and fixed with 4% paraformaldehyde at RT for 20 min. The cells were incubated with mAb YN-2 (FIPV N protein-specific mAb) at 37 °C for 45 min. After washing, the cells were incubated with goat anti-mouse-IgG conjugated to Fluorescein (Jackson ImmunoResearch, PA, USA) at 37 °C for 45 min. After washing, the cells were stained with 4′,6-diamidino-2-phenylindole (DAPI; Dojindo laboratories, Kumamoto, JAPAN) at RT for 30 min. The stained cells were analyzed using Leica DM4B microscope and LAS X integrated imaging system (Leica Microsystems, Wetzlar, Germany).

### 2.6. Statistical Analysis

Data from only two groups were analyzed using the Student’s *t*-test (Welch’s *t*-test) and those of multiple groups were analyzed by one-way ANOVA followed by Tukey’s test using Microsoft Excel 2010 software and open-source statistical GraphPad Prism 8 (GraphPad Software, CA, USA). A *p*-value of <0.05 was considered statically significant.

## 3. Results

### 3.1. Cytotoxic and Antiviral Effects of CQ and HCQ

**Figure 1:**
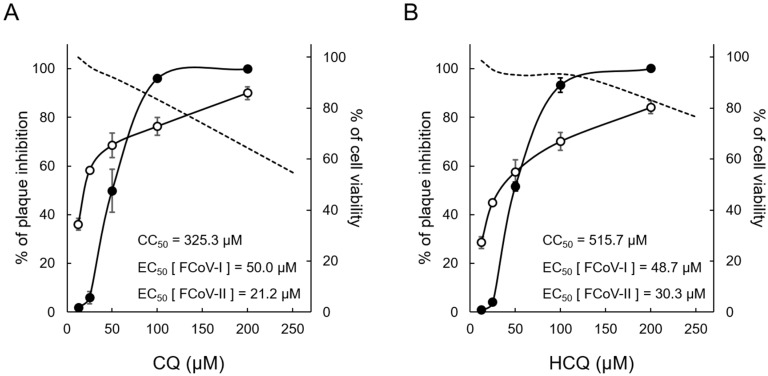
Comparison of the antiviral effects on feline infectious peritonitis virus (FIPV) between chloroquine (CQ) and Hydroxychloroquine (HCQ). CQ and HCQ were separately added to cells at each concentration, and the rate of cell viability was measured after 24 h. In addition, antiviral activities of these drugs were evaluated with regard to the rate of plaque inhibition. (**A**) CQ-treated fcwf-4 cells. (**B**) HCQ-treated fcwf-4 cells. The broken lines represent the rate of cell viability and solid lines represent the rate of plaque inhibition. Black circle: type I FIPV. White circle: type II FIPV. The results are shown as the mean ± SE. Data represent three independent experiments.

Cytotoxicity assay was performed to clarify the non-toxic concentration of CQ and HCQ against fcwf-4 cells (Figure 1). The CC_50_ of CQ and HCQ was 325.3 μM (Figure 1A) and 515.7 μM (Figure 1B), respectively. HCQ was less toxic (36.9%) than CQ in feline cells. The antiviral activity of compounds against both serotypes of FIPV was evaluated using plaque inhibition assay. The EC_50_ of CQ and HCQ against FIPV-I KU2 was 50.0 μM (Figure 1A) and 48.7 μM (Figure 1B), respectively. The EC_50_ of CQ and HCQ against FIPV-II 79-1146 was 21.2 μM (Figure 1A) and 30.3 μM (Figure 1B), respectively. Therefore, the antiviral effects of HCQ on FIPV were comparable to those of CQ. Based on these results, HCQ was suggested to be a safer anti-FIPV drug than CQ.

### 3.2. Long-Term Antiviral Effects of HCQ and rfIFN-ω

**Figure 2:**
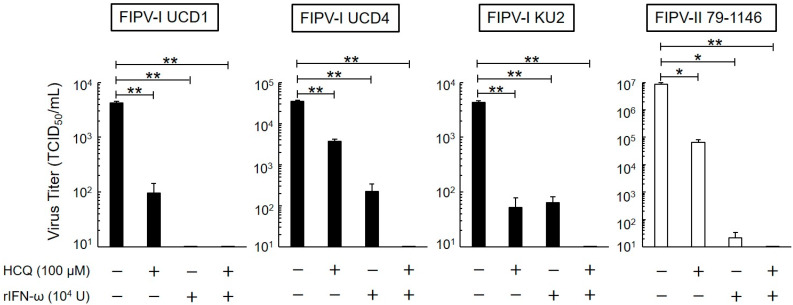
Effects of 24-h exposure on antiviral activity of HCQ and recombinant feline interferon-ω (rfIFN-ω). Virus titer of FIPV-infected fcwf-4 cells pre-treated with HCQ (100 μM) and rfIFN-ω (10^4^ U/mL) for 24 h. The results are shown as the mean ± SE. Data represent four independent experiments. ** *p* < 0.01 (* *p* < 0.05) vs. untreated (pre-treated with medium only).

The antiviral activity of HCQ and rfIFN-ω in fcwf-4 cells for a prolonged time (24 h) was investigated. To evaluate and assess the antiviral effects of HCQ (100 μM) and rfIFN-ω (10^4^ U/mL), we used 3 strains of serotype I FIPV (FIPV-I UCD1, FIPV-I UCD4, and FIPV-I KU2) and 1 strain of serotype II FIPV (FIPV-II 79-1146). The virus titers of both serotypes of FIPV significantly decreased in the culture supernatant of cells pretreated with HCQ or rfIFN-ω (Figure 2). We evaluated the antiviral effects of the combination of HCQ and rfIFN-ω. The combination of these drugs strongly suppressed the replication of viruses in fcwf-4 cells. When both 100 μM HCQ and 10^4^ U/mL of rfIFN-ω were added, fcwf-4 cell viability was 95.6 ± 3.7%.

### 3.3. Short-Term Antiviral Effects of HCQ and rfIFN-ω

**Figure 3:**
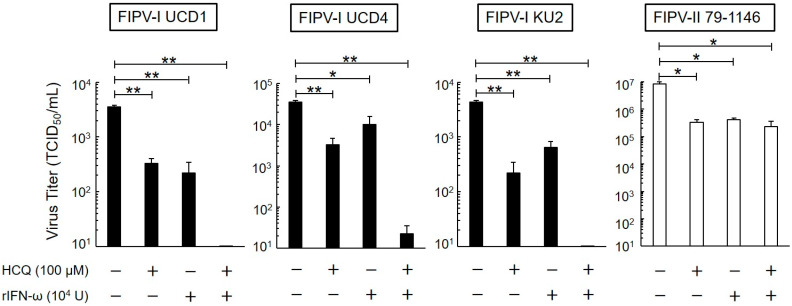
Effects of 1-h exposure on antiviral activity of HCQ and rfIFN-ω. Virus titer of FIPV-infected fcwf-4 cells pre-treated with HCQ (100 μM) and rfIFN-ω (10^4^ U/mL) for 1 h. The results are shown as the mean ± SE. Data represent four independent experiments. ** *p* < 0.01 (* *p* < 0.05) vs. untreated (pre-treated with medium only).

We investigated whether HCQ and rfIFN-ω, which acted on FIPV in fcwf-4 cells for a short time (1 h), exhibit antiviral activity. As shown in Figure 3, type I FIPV and type II FIPV replication was significantly inhibited by HCQ and rfIFN-ω. Interestingly, the combination of these drugs strongly decreased the replication of type I FIPVs in fcwf-4 cells, but not type II FIPV.

### 3.4. Post-Treatment Antiviral Effects of HCQ and rfIFN-ω

**Figure 4:**
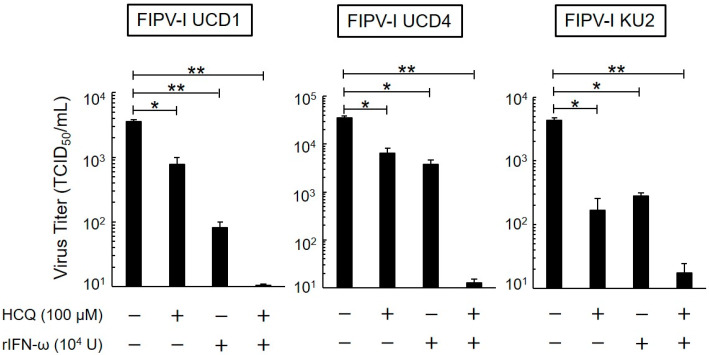
Effects of post-exposure on antiviral activity of HCQ and rfIFN-ω. Virus titer of type I FIPV-infected fcwf-4 cells post-treated with HCQ (100 μM) and rfIFN-ω (10^4^ U/mL). The results are shown as the mean ± SE. Data represent four independent experiments. ** *p* < 0.01 (* *p* < 0.05) vs. untreated (post-treated with medium only).

We evaluated the antiviral activity of HCQ and rfIFN-ω against type I FIPV after viral infection. HCQ and rfIFN-ω were added to the cells 1 h after inoculation. As shown in Figure 4, type I FIPV replication was significantly inhibited by HCQ and rfIFN-ω, and the combination of these drugs strongly decreased the replication of virus.

### 3.5. Effects of HCQ and rfIFN-ω on FIPV N Protein Expression

**Figure 5:**
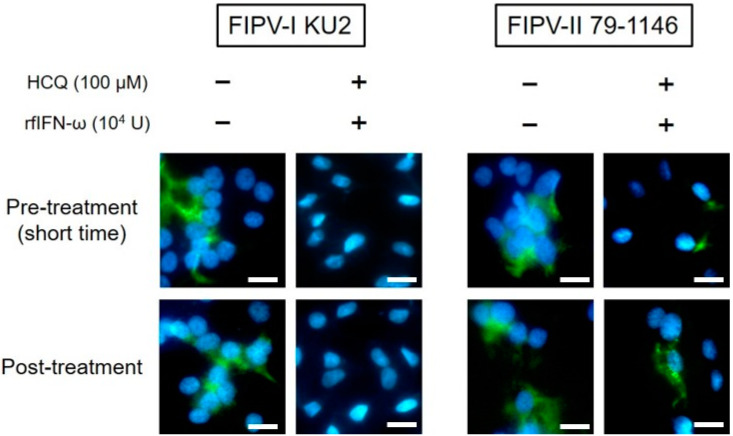
Effects of HCQ and rfIFN-ω on FIPV N protein expression. FIPV N protein was evaluated by IFA. Scale bar = 10 μm.

We investigated the expression of viral proteins in order to evaluate the antiviral effects of the combination of HCQ and rfIFN-ω on FIPV. The N protein levels of FIPV-I KU-2 were specifically decreased in fcwf-4 cells pre-treated (short-time exposure) and post-treated with HCQ and rfIFN-ω (Figure 5). In contrast, post-treatment with HCQ and rfIFN-ω slightly affected the protein levels of FIPV-II 79-1146 in fcwf-4 cells.

## 4. Discussion

FIP is a fatal coronaviral infection of cats. Several drugs have been identified aiming at the treatment of FIP, but no commercial drugs can be used to treat FIP by veterinarians. We have searched for a drug applicable to treat FIP among commercial drugs [24,25]. CQ is an antimalarial drug and improved symptoms of cats with FIP [11]. However, increased liver enzymes were observed in some cats treated with CQ. Increased liver enzymes are observed in cats with FIP, but the possibility of CQ-induced liver disorder was also suggested. If there is a drug with cytotoxicity weaker than that of CQ that exhibits comparable antiviral effects, it may be applicable as a therapeutic drug for FIP. We focused on HCQ, which is 4-aminoquinoline similar to CQ [26]. The cytotoxicity of HCQ has been reported to be lower than that of CQ in mouse, rat, and dog [12]. In addition, HCQ has been demonstrated to have antiviral effects on SARS-CoV-2 infection equivalent to those of CQ in vitro [13].

We confirmed that HCQ has anti-FIPV activity equivalent to that of CQ. Moreover, cytotoxicity of HCQ setting the criterion to CC_50_ was one-third or lower than that of CQ. Accordingly, HCQ is applicable to FIP treatment as a substitute for CQ. HCQ at 100 μM significantly inhibited the replication of both serotypes of FIPV. To our knowledge, the pharmacokinetics of HCQ in cats have not been analyzed. Thus, it is necessary to refer to pharmacokinetic data of HCQ in dogs. The tolerated dose of intramuscular injection of HCQ is 25 mg/kg [12]. In dogs treated with 25 mg/kg of HCQ, the plasma HCQ level reaches 3.23 μM (1400 μg/L) [12], i.e., it is difficult to make the plasma HCQ level reach 100 μM in dogs. However, it has been reported that the tissue HCQ levels in the liver, spleen, kidney, and lung increased to a level several hundred-times higher than the plasma level [13]. Therefore, HCQ administration to cats with FIP within the low dosage may be expected to yield sufficient therapeutic effects. On the other hand, it is unclear whether the pharmacokinetics described above can apply to cats. Cytochrome P450 (CYPs) are involved in the metabolism of HCQ [27]. Generally, CYP activities could be lower in cats than in dogs [28]. On the basis of this fact, the blood concentrations of HCQ in cats will be higher than those in dogs. Therefore, pharmacokinetic studies are still needed to use HCQ in cats.

The antiviral agent rfIFN-ω has a wide safety range and is practically used to treat feline viral infection in veterinary practice. Many points are unclear as to whether rfIFN-ω is effective as a therapeutic drug for FIP.

The combination of HCQ and rfIFN-ω blocked virus production in type I FIPV-infected cells, but although the duration of activity was only 1 h, the antiviral activity of these drugs decreased in type II FIPV-infected cells. We previously demonstrated that types I and II FIPV enter the cytosol through late and early endosomes, respectively [29]. We also reported that type II FIPV strongly inhibited type I IFN expression [30]. Based on this knowledge and our current study, type II FIPV may show less effect on the antiviral activity of HCQ and type I IFN, compared to type I FIPV.

There are some reports about the relationship between HCQ and type I IFN. Wang et al. reported that HCQ inhibited dengue virus infection in all serotypes in vitro [14]. They suggested that the induction of interferon or related protein is an antiviral activity mechanism of HCQ. On the other hand, inhibition of type I IFN production in HCQ-treated cells has been reported [31], being contradictory to other findings. We confirmed that potent antiviral activity was induced by the combination of HCQ and rfIFN-ω (type I IFN). Although negative action on type I IFN may have been induced by HCQ, type I IFN added at the same time may have canceled this. To demonstrate this, further investigation is necessary.

In this study, we confirmed that HCQ is a safer anti-FIPV drug than CQ. In addition, we demonstrated that the combination of HCQ and rfIFN-ω increases the antiviral activity. Our study revealed that these FIP therapeutic drugs are applicable to veterinary practice. It should be noted that in vitro data do not always translate into in vivo efficacy. Therefore, a deeper understanding of pharmacokinetics of the combination between HCQ and rfIFN-ω will be needed in cats.
